# Trends in the Prevalence and Severity of Alcohol and Cannabis Use Disorders Among US Adults

**DOI:** 10.1001/jamapsychiatry.2026.2497

**Published:** 2026-08-19

**Authors:** Beth Han, Wilson M. Compton, Jennifer A. Hobin, Nora D. Volkow

**Affiliations:** 1 National Institute on Drug Abuse, National Institutes of Health, Bethesda, Maryland

## Abstract

**Importance:**

The number of US adults with alcohol use disorder (AUD) has declined, whereas the number with cannabis use disorder (CUD) has increased. Little is known about how changes in the prevalence and severity of AUD and CUD vary by age and sex.

**Objective:**

To investigate trends in AUD and CUD prevalence and severity by age and sex.

**Design, Setting, and Participants:**

This cross-sectional study examined data from adult participants in the 2021 to 2024 National Surveys on Drug Use and Health. This study was started and completed in March 2026.

**Exposure:**

Alcohol and cannabis use.

**Main Outcomes and Measures:**

Past-year prevalence and severity of AUD and CUD using *DSM-5* criteria.

**Results:**

From 2021 through 2024 among 186 823 participants (51.3% female; 4.9% aged 18-20 years; 24.1% aged 21-34 years; 24.7% aged 35-49 years), past-year CUD prevalence increased among males (from 7.3% [95% CI, 6.6%-8.1%] to 9.3% [95% CI, 8.6%-10.1%]; *P* < .001) and females (from 4.5% [95% CI, 4.1%-5.0%] to 5.6% [95% CI, 5.2%-6.1%]; *P* = .01). Moderate-to-severe CUD prevalence among males increased from 3.1% (95% CI, 2.8%-3.6%) to 4.1% (95% CI, 3.7%-4.5%) (*P* < .001), especially among males aged 35 to 49 years (from 2.7% [95% CI, 2.0%-3.6%] to 3.9% [95% CI, 3.2%-4.8%]; *P* = .01) and males aged 50 years or older (from 0.6% [95% CI, 0.3%-1.0%] to 1.2% [95% CI, 0.8%-1.7%]; *P* = .02). Among females, moderate-to-severe CUD prevalence increased from 1.7% (95% CI, 1.5%-2.0%) to 2.5% (95% CI, 2.3%-2.8%) (*P* < .001), specifically for females aged 21 to 34 years (from 4.2% [95% CI, 3.5%-5.1%] to 5.6% [95% CI, 4.8%-6.5%]; *P* = .04), 35 to 49 years (from 1.2% [95% CI, 0.8%-1.8%] to 2.0% [95% CI, 1.6%-2.5%]; *P* = .01), and 50 years or older (from 0.1% [95% CI, 0.04%-0.3%] to 0.7% [95% CI, 0.5%-1.0%]; *P* < .001). Among females with CUD, moderate-to-severe CUD prevalence increased from 37.0% (95% CI, 32.5%-41.8%) to 45.0% (95% CI, 41.1%-49.0%) (*P* = .03). However, moderate-to-severe AUD prevalence remained stable among males overall (except for declines among males aged 21-34 years) and was stable among females overall and within all subgroups. Most males and females aged 18 to 20 years and males aged 21 to 34 years with CUD had moderate-to-severe CUD (eg, 51.0%-61.0% in 2024), with consistent patterns from 2022 through 2024.

**Conclusions and Relevance:**

This cross-sectional study found divergent age- and sex-specific patterns from 2021 through 2024, with moderate-to-severe CUD prevalence increasing and moderate-to-severe AUD prevalence remaining stable across males, females, and most subgroups. The increased prevalence underscores the importance of early detection, routine screening, timely access to evidence-based CUD treatment, and targeted strategies to counter increasing acceptance, availability, and potency of cannabis products. High prevalence of moderate-to-severe CUD among adults who use cannabis challenges the notion that cannabis has low addictive potential.

## Key Points

**Question:**

What are patterns of change in prevalence and severity of alcohol use disorder (AUD) and cannabis use disorder (CUD) as a function of age and sex?

**Findings:**

In this cross-sectional study, divergent age- and sex-specific patterns emerged from 2021 through 2024: CUD and moderate-to-severe CUD prevalence increased, while AUD and moderate-to-severe AUD prevalence remained stable or declined. Moderate-to-severe CUD increased among males (from 3.1% to 4.1%, especially in males aged 35-49 and ≥50 years) and females (from 1.7% to 2.5%, especially in females aged 21-34, 35-49, and ≥50 years).

**Meaning:**

Moderate-to-severe CUD warrants clinical treatment, and significant increases in its prevalence challenge the notion that cannabis has low addictive potential.

## Teaser

This cross-sectional study investigates age- and sex-specific trends in the prevalence and severity of alcohol and cannabis use disorders among US adults.

## Introduction

Although alcohol use disorder (AUD) remains a highly prevalent substance use disorder,^1,2^ the number of US adults with AUD declined from 28.8 million to 27.1 million from 2021 through 2024.^3^ By contrast, the number of adults with cannabis use disorder (CUD) increased from 15.3 million to 19.4 million from 2021 through 2024,^3^ paralleling increasing cannabis potency, expanding legalization,^2,4,5^ and decreasing perceived harm.^6^ To inform public health prevention and treatment strategies, we investigated patterns of changes in AUD and CUD prevalence and severity by age and sex, with particular attention to the growing divergence between these 2 substance use disorders.

## Methods

In this cross-sectional study, data were from 186 823 participants aged 18 years or older in the 2021 through 2024 National Surveys on Drug Use and Health (NSDUH), providing nationally representative data on cannabis and alcohol use, CUD, and AUD (using *DSM-5* criteria beginning in 2021; moderate-to-severe disorder was indicated by ≥4 symptoms) among US civilian, noninstitutionalized adult populations.^3^ The RTI International institutional review board approved the NSDUH data collection protocols. Participants provided written or verbal informed consent.^3^ This study used publicly available deidentified data and was exempt from National Institutes of Health institutional review board review. Logistic regression analyses (*P* < .05, 2-sided) were conducted using SUDAAN release 11.0.1 (RTI International) to account for NSDUH’s complex sample design and sampling weights. The Strengthening the Reporting of Observational Studies in Epidemiology (STROBE) reporting guideline for cross-sectional studies was followed.

## Results

This cross-sectional study evaluated data from 186 823 participants (51.3% female) who completed the National Survey on Drug Use and Health from 2021 through 2024. Among them, 4.9% were aged 18 to 20 years; 24.1%, 21 to 34 years; and 24.7%, 35 to 49 years.

### CUD Prevalence and Severity Among Males by Age

Among males aged 18 years older from 2021 through 2024, past-year CUD prevalence increased from 7.3% (95% CI, 6.6%-8.1%) to 9.3% (95% CI, 8.6%-10.1%) (*P* < .001) (Table 1), paralleling increases in cannabis use (from 22.2% [95% CI, 20.8%-23.8%] to 25.7% [95% CI, 24.7%-26.9%]; *P* = .001) (Table 2) and CUD prevalence among those who used cannabis (from 32.9% [95% CI, 30.5%-35.3%] to 36.2% [95% CI, 34.0%-38.5%]; *P* = .02) (Table 3). CUD prevalence increased from 41.6% (95% CI, 35.5%-48.0%) to 50.0% (95% CI, 44.2%-55.9%) (*P* = .03) among 18- to 20-year-old males using cannabis; from 7.2% (95% CI, 5.7%-9.0%) to 10.4% (95% CI, 9.1%-11.9%) (*P* < .001) among 35- to 49-year-old males, coinciding with increases in cannabis use (from 22.5% [95% CI, 19.9%-25.2%] to 29.1% [95% CI, 27.3%-31.0%]; *P* < .001); and from 2.4% (95% CI, 1.8%-3.3%) to 3.8% (95% CI, 3.0%-4.8%) (*P* = .01) among males aged 50 years or older, with corresponding increases in cannabis use (from 13.4% [95% CI, 11.7%-15.3%] to 16.9% [95% CI, 15.2%-18.9%]; *P* = .008). Moderate-to-severe CUD prevalence among males aged 18 years or older increased from 3.1% (95% CI, 2.8%-3.6%) to 4.1% (95% CI, 3.7%-4.5%) (*P* < .001), especially among those aged 35 to 49 years (from 2.7% [95% CI, 2.0%-3.6%] to 3.9% [95% CI, 3.2%-4.8%]; *P* = .01) and 50 years or older (0.6% [95% CI, 0.3%-1.0%] to 1.2% [95% CI, 0.8%-1.7%]; *P* = .02).

**Table 1.** Trends in Past-Year Prevalence and Severity of Cannabis Use Disorder (CUD) and Alcohol Use Disorder (AUD) Among US Adults by Age and Sex^a^

Past-year prevalence	Weighted % (95% CI) by year				Trend	
	2021	2022	2023	2024	β	*P* value
**Overall, aged ≥18 y**						
CUD in males	7.3 (6.6-8.1)	8.5 (7.8-9.3)	8.6 (8.0-9.2)	9.3 (8.6-10.1)	0.08	<.001
MS CUD in males	3.1 (2.8-3.6)	3.8 (3.4-4.2)	3.8 (3.5-4.1)	4.1 (3.7-4.5)	0.08	<.001
CUD in females	4.5 (4.1-5.0)	5.5 (5.1-6.0)	5.3 (5.0-5.7)	5.6 (5.2-6.1)	0.06	.01
MS CUD in females	1.7 (1.5-2.0)	2.4 (2.1-2.7)	2.3 (2.0-2.6)	2.5 (2.3-2.8)	0.11	<.001
AUD in males	13.3 (12.4-14.2)	13.8 (13.1-14.6)	13.2 (12.5-14.0)	12.6 (11.9-13.3)	−0.02	.13
MS AUD in males	5.8 (5.2-6.3)	6.0 (5.5-6.6)	5.4 (5.0-6.0)	5.2 (4.7-5.7)	−0.04	.08
AUD in females	9.7 (8.9-10.6)	8.9 (8.4-9.4)	8.7 (8.1-9.3)	8.2 (7.7-8.8)	−0.06	.02
MS AUD in females	3.7 (3.2-4.4)	3.3 (2.9-3.7)	3.7 (3.4-4.1)	3.2 (2.9-3.6)	−0.03	.28
**Aged 18-20 y**						
CUD in males	12.3 (10.1-15.0)	14.8 (12.8-17.2)	17.0 (14.4-19.9)	14.9 (12.9-17.2)	0.08	.06
MS CUD in males	6.8 (5.2-8.9)	9.6 (7.6-12.1)	9.0 (7.4-11.0)	7.7 (6.1-9.6)	0.03	.63
CUD in females	14.5 (11.7-17.7)	15.1 (12.9-17.6)	13.2 (11.5-15.0)	14.5 (12.6-16.5)	−0.02	.72
MS CUD in females	7.2 (5.4-9.7)	9.4 (7.9-11.1)	6.7 (5.3-8.4)	8.8 (7.3-10.6)	0.03	.60
AUD in males	6.9 (5.2-9.2)	11.1 (9.1-13.5)	10.4 (8.6-12.6)	10.4 (8.4-12.8)	0.11	.03
MS AUD in males	2.5 (1.5-4.1)	4.8 (3.6-6.4)	4.1 (3.0-5.5)	4.1 (3.0-5.7)	0.12	.09
AUD in females	13.0 (10.7-15.7)	10.4 (8.7-12.5)	11.4 (8.9-14.4)	10.3 (8.7-12.2)	−0.07	.14
MS AUD in females	4.9 (3.5-6.7)	3.9 (2.8-5.5)	5.6 (4.0-7.8)	4.6 (3.6-6.0)	−0.02	.73
**Aged 21-34 y**						
CUD in males	15.4 (13.7-17.3)	16.9 (15.4-18.6)	15.4 (14.4-16.5)	17.1 (15.9-18.4)	0.03	.25
MS CUD in males	7.6 (6.4-9.0)	8.8 (7.9-9.8)	8.2 (7.4-9.1)	8.7 (7.9-9.7)	0.04	.24
CUD in females	9.7 (8.5-11.0)	12.0 (11.0-13.1)	11.1 (10.2-12.1)	11.5 (10.6-12.4)	0.05	.11
MS CUD in females	4.2 (3.5-5.1)	5.6 (5.0-6.2)	5.1 (4.5-5.8)	5.6 (4.8-6.5)	0.07	.04
AUD in males	20.0 (18.3-21.8)	19.9 (18.4-21.6)	18.5 (17.0-20.1)	17.0 (15.8-18.2)	−0.07	.004
MS AUD in males	9.4 (8.2-10.8)	9.7 (8.6-10.8)	7.9 (6.8-9.2)	6.8 (6.0-7.7)	−0.12	<.001
AUD in females	15.0 (13.7-16.5)	15.2 (14.0-16.5)	15.0 (13.7-16.4)	12.6 (11.5-13.8)	−0.06	.02
MS AUD in females	5.9 (4.8-7.2)	6.5 (5.5-7.6)	7.3 (6.4-8.3)	5.1 (4.5-5.8)	−0.03	.36
**Aged 35-49 y**						
CUD in males	7.2 (5.7-9.0)	8.8 (7.4-10.4)	8.8 (7.7-10.0)	10.4 (9.1-11.9)	0.12	<.001
MS CUD in males	2.7 (2.0-3.6)	3.0 (2.3-3.9)	3.3 (2.7-4.2)	3.9 (3.2-4.8)	0.13	.01
CUD in females	3.8 (3.1-4.7)	5.2 (4.4-6.2)	5.3 (4.6-6.2)	5.6 (4.8-6.5)	0.11	.004
MS CUD in females	1.2 (0.8-1.8)	1.7 (1.3-2.3)	2.1 (1.7-2.5)	2.0 (1.6-2.5)	0.16	.01
AUD in males	15.1 (13.6-16.6)	15.7 (13.9-17.8)	14.8 (13.2-16.6)	15.7 (14.2-17.4)	0.01	.74
MS AUD in males	6.6 (5.6-7.8)	6.4 (5.5-7.6)	6.6 (5.4-8.0)	7.7 (6.4-9.2)	0.05	.18
AUD in females	11.1 (9.9-12.3)	9.9 (8.7-11.3)	10.1 (9.0-11.4)	8.9 (7.8-10.1)	−0.07	.04
MS AUD in females	4.8 (4.0-5.8)	4.0 (3.4-4.8)	4.0 (3.3-4.7)	3.8 (3.1-4.5)	−0.05	.10
**Aged ≥50 y**						
CUD in males	2.4 (1.8-3.3)	3.0 (2.2-4.0)	3.9 (2.9-5.1)	3.8 (3.0-4.8)	0.16	.01
MS CUD in males	0.6 (0.3-1.0)	0.8 (0.5-1.4)	1.0 (0.6-1.4)	1.2 (0.8-1.7)	0.23	.02
CUD in females	1.4 (0.8-2.4)	1.4 (1.0-2.0)	1.7 (1.3-2.3)	1.9 (1.4-2.5)	0.12	.25
MS CUD in females	0.1 (0.04-0.3)	0.5 (0.3-0.9)	0.6 (0.3-1.1)	0.7 (0.5-1.0)	0.40	<.001
AUD in males	9.5 (8.1-11.1)	9.7 (8.3-11.4)	9.7 (8.6-11.1)	8.6 (7.5-9.8)	−0.03	.34
MS AUD in males	3.7 (3.0-4.7)	3.9 (3.0-5.1)	3.6 (2.8-4.6)	3.0 (2.4-3.8)	−0.07	.17
AUD in females	6.1 (4.8-7.6)	5.0 (4.3-5.9)	4.5 (3.8-5.4)	5.5 (4.7-6.5)	−0.04	.48
MS AUD in females	2.0 (1.3-3.1)	1.3 (1.0-1.6)	1.7 (1.3-2.2)	1.8 (1.4-2.4)	−0.003	.97

Abbreviation: MS, moderate-to-severe.

^a^ Data sources were the 2021 through 2024 National Surveys on Drug Use and Health public-use data.

**Table 2.** Trends in Past-Year Prevalence of Cannabis and Alcohol Use Among US Adults by Age and Sex^a^

Past-year prevalence	Weighted % (95% CI) by year				Trend	
	2021	2022	2023	2024	β	*P* value
**Overall, aged ≥18 y**						
Cannabis use in males	22.2 (20.8-23.8)	25.8 (24.8-26.9)	25.7 (24.7-26.8)	25.7 (24.7-26.9)	0.06	.001
Cannabis use in females	17.7 (16.9-18.6)	20.2 (19.3-21.2)	20.4 (19.6-21.2)	21.2 (20.2-22.2)	0.07	<.001
Alcohol use in males	69.0 (67.6-70.4)	69.3 (68.2-70.4)	69.3 (68.3-70.2)	67.3 (66.0-68.6)	−0.02	.09
Alcohol use in females	64.9 (63.4-66.3)	65.3 (63.9-66.7)	64.9 (63.5-66.3)	65.3 (64.0-66.5)	0.004	.79
**Aged 18-20 y**						
Cannabis use in males	29.7 (26.5-33.0)	34.2 (31.2-37.4)	33.8 (31.0-36.7)	29.8 (26.7-33.1)	0.001	.98
Cannabis use in females	33.4 (29.4-37.7)	35.1 (31.6-38.8)	32.2 (29.3-35.3)	30.7 (28.0-33.5)	−0.05	.14
Alcohol use in males	49.7 (46.4-53.2)	50.8 (47.0-54.5)	49.5 (45.8-53.3)	47.7 (43.8-51.7)	−0.03	.31
Alcohol use in females	52.3 (48.2-56.4)	51.8 (48.5-55.1)	51.9 (48.4-55.3)	50.5 (47.3-53.6)	−0.02	.46
**Aged 21-34 y**						
Cannabis use in males	37.0 (34.4-39.7)	40.8 (38.7-42.9)	38.0 (36.2-39.9)	37.4 (35.6-39.3)	−0.01	.75
Cannabis use in females	30.7 (28.9-32.7)	34.5 (32.8-36.3)	33.7 (32.0-35.4)	33.5 (32.0-35.2)	0.03	.07
Alcohol use in males	77.3 (75.5-78.9)	77.3 (75.8-78.7)	77.3 (75.7-78.8)	75.6 (74.3-76.9)	−0.03	.15
Alcohol use in females	76.3 (74.3-78.2)	76.3 (74.9-77.6)	76.4 (74.6-78.1)	75.4 (73.7-77.1)	−0.01	.56
**Aged 35-49 y**						
Cannabis use in males	22.5 (19.9-25.2)	26.9 (24.7-29.2)	28.5 (26.3-30.8)	29.1 (27.3-31.0)	0.11	<.001
Cannabis use in females	18.9 (17.3-20.7)	21.9 (20.5-23.3)	22.8 (21.3-24.3)	23.7 (22.2-25.3)	0.09	<.001
Alcohol use in males	72.3 (69.1-75.3)	73.0 (70.8-75.1)	72.4 (70.6-74.1)	72.1 (70.2-73.8)	−0.01	.79
Alcohol use in females	70.2 (67.5-72.7)	71.1 (69.4-72.7)	69.3 (67.2-71.3)	69.4 (67.5-71.3)	−0.02	.42
**Aged ≥50 y**						
Cannabis use in males	13.4 (11.7-15.3)	16.0 (14.3-17.9)	16.5 (15.1-18.1)	16.9 (15.2-18.9)	0.08	.008
Cannabis use in females	9.1 (7.9-10.5)	10.8 (9.3-12.4)	11.4 (10.2-12.8)	12.8 (11.6-14.0)	0.12	<.001
Alcohol use in males	65.2 (62.6-67.6)	65.2 (63.2-67.0)	65.3 (63.7-67.0)	62.2 (59.9-64.4)	−0.04	.08
Alcohol use in females	57.8 (55.4-60.1)	58.2 (55.8-60.6)	58.2 (55.7-60.7)	59.5 (57.6-61.3)	0.02	.30

^a^ Data sources were the 2021 through 2024 National Surveys on Drug Use and Health public-use data.

**Table 3.** Trends in Prevalence and Severity of Cannabis Use Disorder (CUD) and Alcohol Use Disorder (AUD) Among Those Who Used the Substances in the Past Year, by Age and Sex^a^

Past-year prevalence	Weighted % (95% CI) by year				Trend	
	2021	2022	2023	2024	β	*P* value
**Overall, aged ≥18 y**						
CUD in males using cannabis	32.9 (30.5-35.3)	33.0 (30.8-35.2)	33.5 (31.5-35.6)	36.2 (34.0-38.5)	0.05	.02
MS in those with CUD	43.0 (38.7-47.4)	44.6 (41.1-48.1)	43.7 (40.7-46.6)	43.7 (41.1-46.3)	0.003	.93
CUD in females using cannabis	25.6 (23.3-28.1)	27.2 (25.3-29.1)	26.2 (24.5-27.9)	26.6 (24.6-28.7)	0.01	.76
MS in those with CUD	37.0 (32.5-41.8)	43.9 (39.6-48.4)	42.7 (38.6-46.9)	45.0 (41.1-49.0)	0.09	.03
AUD in males using alcohol	19.2 (18.0-20.6)	20.0 (18.8-21.1)	19.1 (18.0-20.1)	18.7 (17.6-19.7)	−0.02	.30
MS in those with AUD	43.3 (40.3-46.4)	43.5 (40.5-46.5)	41.2 (38.0-44.5)	41.4 (38.5-44.4)	−0.03	.24
AUD in females using alcohol	15.0 (13.7-16.3)	13.6 (12.9-14.3)	13.4 (12.6-14.2)	12.6 (11.8-13.5)	−0.06	.01
MS in those with AUD	38.5 (34.6-42.6)	37.1 (33.5-40.8)	43.1 (40.2-46.1)	38.9 (35.7-42.2)	0.03	.32
**Aged 18-20 y**						
CUD in males using cannabis	41.6 (35.5-48.0)	43.4 (38.3-46.7)	50.2 (43.3-57.0)	50.0 (44.2-55.9)	0.13	.03
MS in those with CUD	55.5 (45.4-65.1)	64.5 (56.3-72.0)	53.2 (46.5-60.0)	51.5 (42.0-60.9)	−0.10	.28
CUD in females using cannabis	43.4 (35.6-51.4)	43.1 (38.8-47.6)	41.0 (36.1-46.0)	47.2 (42.2-52.2)	0.04	.57
MS in those with CUD	50.0 (40.2-59.8)	62.1 (53.3-70.2)	50.7 (40.4-61.0)	61.0 (53.3-68.2)	0.09	.28
AUD in males using alcohol	13.9 (10.6-18.0)	21.9 (18.3-26.0)	21.0 (17.4-25.2)	21.7 (18.0-26.0)	0.15	.01
MS in those with AUD	36.1 (23.7-50.6)	43.3 (34.8-52.3)	38.9 (30.2-48.4)	40.0 (29.5-51.4)	0.02	.87
AUD in females using alcohol	24.8 (20.7-29.4)	20.1 (16.7-23.9)	21.9 (17.4-27.3)	20.5 (17.5-23.8)	−0.07	.21
MS in those with AUD	37.4 (28.1-47.8)	37.6 (27.8-48.7)	49.2 (38.9-59.5)	45.0 (34.7-55.8)	0.14	.16
**Aged 21-34 y**						
CUD in males using cannabis	41.7 (38.2-45.2)	41.5 (38.5-44.6)	40.6 (38.1-43.2)	45.8 (42.9-48.7)	0.05	.08
MS in those with CUD	49.3 (43.5-55.1)	51.9 (47.5-56.4)	53.3 (49.5-57.1)	51.0 (47.1-54.9)	0.02	.62
CUD in females using cannabis	31.4 (28.0-35.1)	34.9 (32.2-37.6)	33.0 (30.6-35.5)	34.2 (31.7-36.8)	0.03	.41
MS in those with CUD	43.8 (37.7-50.1)	46.1 (42.3-50.0)	46.1 (41.7-50.6)	48.7 (43.8-53.7)	0.06	.19
AUD in males using alcohol	25.9 (23.8-28.1)	25.8 (24.0-27.8)	23.9 (22.1-25.9)	22.4 (21.0-23.9)	−0.07	.01
MS in those with AUD	47.1 (42.9-51.3)	48.4 (44.3-52.6)	42.9 (38.1-47.9)	40.1 (36.2-44.3)	−0.11	.009
AUD in females using alcohol	19.7 (18.0-21.5)	19.9 (18.4-21.6)	19.6 (18.0-21.3)	16.7 (15.2-18.4)	−0.06	.03
MS in those with AUD	39.3 (33.4-45.5)	42.4 (36.9-48.2)	48.6 (44.5-52.7)	40.4 (36.6-44.4)	0.05	.28
**Aged 35-49 y**						
CUD in males using cannabis	32.1 (26.8-37.8)	32.7 (38.4-37.3)	30.7 (27.2-34.5)	35.7 (32.2-39.4)	0.05	.26
MS in those with CUD	37.3 (29.4-45.9)	33.6 (26.4-41.8)	38.1 (31.9-44.7)	37.6 (32.6-42.9)	0.03	.66
CUD in females using cannabis	20.4 (16.6-24.7)	23.8 (20.2-27.8)	23.4 (20.2-26.9)	23.6 (20.4-27.1)	0.05	.29
MS in those with CUD	31.5 (21.8-43.1)	33.5 (26.3-41.7)	38.6 (32.1-45.6)	35.8 (29.9-42.1)	0.07	.36
AUD in males using alcohol	20.8 (18.8-23.0)	21.5 (19.0-24.4)	20.4 (18.2-22.8)	21.8 (19.6-24.1)	0.01	.67
MS in those with AUD	43.8 (37.6-50.2)	41.0 (35.3-47.0)	44.5 (38.3-50.9)	48.9 (42.5-55.3)	0.08	.19
AUD in females using alcohol	15.8 (14.2-17.5)	13.9 (12.2-15.8)	14.6 (13.1-16.3)	12.8 (11.2-14.5)	−0.07	.05
MS in those with AUD	43.5 (38.1-49.1)	40.5 (35.6-45.7)	39.0 (33.5-44.9)	42.5 (37.2-48.0)	−0.02	.65
**Aged ≥50 y**						
CUD in males using cannabis	18.2 (13.6-24.0)	18.5 (14.1-24.0)	23.4 (18.0-29.7)	22.2 (17.9-27.3)	0.10	.14
MS in those with CUD	23.4 (13.0-38.6)	27.5 (16.2-42.7)	24.9 (17.3-34.6)	31.4 (22.9-41.4)	0.11	.42
CUD in females using cannabis	14.9 (8.8-24.2)	13.3 (9.7-18.0)	15.1 (11.6-19.4)	14.7 (11.1-19.1)	0.01	.92
MS in those with CUD	7.4 (3.3-15.5)^b^	35.4 (21.0-53.1)^b^	32.5 (17.4-52.3)^b^	35.9 (23.9-49.9)	0.40	.004^b^
AUD in males using alcohol	14.6 (12.5-17.0)	14.9 (12.8-17.4)	14.9 (13.1-16.9)	13.8 (12.0-15.7)	−0.02	.59
MS in those with AUD	39.4 (32.7-46.4)	40.2 (32.5-48.4)	36.9 (29.9-44.5)	35.1 (29.0-41.8)	−0.07	.28
AUD in females using alcohol	10.5 (8.4-13.1)	8.6 (7.4-10.0)	7.8 (6.6-9.1)	9.3 (7.9-10.9)	−0.05	.36
MS in those with AUD	33.1 (23.1-45.0)	25.4 (20.6-30.8)	37.4 (29.3-46.3)	33.3 (25.6-42.0)	0.05	.63

Abbreviation: MS, moderate-to-severe.

^a^ Data sources were the 2021 through 2024 National Surveys on Drug Use and Health public-use data.

^b^ Interpretation should be cautious given the low statistical precision.

### AUD Prevalence and Severity Among Males by Age

Among males aged 18 years or older from 2021 through 2024, AUD and moderate-to-severe AUD prevalence remained stable. For 18- to 20-year-old males, AUD increased from 6.9% (95% CI, 5.2%-9.2%) to 10.4% (95% CI, 8.4%-12.8%) (*P* = .03), paralleling increases in AUD prevalence among those who used alcohol (from 13.9% [95% CI, 10.6%-18.0%] to 21.7% [95% CI, 18.0%-26.0%]; *P* = .01). For 21- to 34-year-old males, AUD declined from 20.0% (95% CI, 18.3%-21.8%) to 17.0% (95% CI, 15.8%-18.2%) (*P* = .004), coinciding with decreased AUD prevalence among those who used alcohol (from 25.9% [95% CI, 23.8%-28.1%] to 22.4% [95% CI, 21.0%-23.9%]; *P* = .01), and moderate-to-severe AUD declined from 9.4% (95% CI, 8.2%-10.8%) to 6.8% (95% CI, 6.0%-7.7%) (*P* < .001), as moderate-to-severe AUD among those with AUD declined (from 47.1% [95% CI, 42.9%-51.3%] to 40.1% [95% CI, 36.2%-44.3%]; *P* = .009).

### CUD Prevalence and Severity Among Females by Age

Among females aged 18 years or older from 2021 through 2024, CUD prevalence increased from 4.5% (95% CI, 4.1%-5.0%) to 5.6% (95% CI, 5.2%-6.1%) (*P* = .01), coinciding with an increase in cannabis use (from 17.7% [95% CI, 16.9%-18.6%] to 21.2% [95% CI, 20.2%-22.2%]; *P* < .001). Among females aged 35 to 49 years, CUD prevalence increased from 3.8% (95% CI, 3.1%-4.7%) to 5.6% (95% CI, 4.8%-6.5%) (*P* = .004), paralleling increases in cannabis use (from 18.9% [95% CI, 17.3%-20.7%] to 23.7% [95% CI, 22.2%-25.3%]; *P* < .001).

Moderate-to-severe CUD prevalence among females aged 18 years or older increased from 1.7% (95% CI, 1.5%-2.0%) to 2.5% (95% CI, 2.3%-2.8%) (*P* < .001), specifically among those aged 21 to 34 years (from 4.2% [95% CI, 3.5%-5.1%] to 5.6% [95% CI, 4.8%-6.5%]; *P* = .04), 35 to 49 years (from 1.2% [95% CI, 0.8%-1.8%] to 2.0% [95% CI, 1.6%-2.5%]; *P* = .01), and 50 years or older (from 0.1% [95% CI, 0.04%-0.3%] to 0.7% [95% CI, 0.5%-1.0%]; *P* < .001). Among females aged 18 years or older with CUD, moderate-to-severe CUD increased from 37.0% (95% CI, 32.5%-41.8%) to 45.0% (95% CI, 41.1%-49.0%) (*P* = .03).

### AUD Prevalence and Severity Among Females by Age

AUD prevalence declined among females aged 18 years or older (from 9.7% [95% CI, 8.9%-10.6%] to 8.2% [95% CI, 7.7%-8.8%]; *P* = .02), especially among those aged 21 to 34 years (from 15.0% [95% CI, 13.7%-16.5%] to 12.6% [95% CI, 11.5%-13.8%]; *P* = .02) and 35 to 49 years (from 11.1% [95% CI, 9.9%-12.3%] to 8.9% [95% CI, 7.8%-10.1%]; *P* = .04). AUD decreased among 21- to 34-year-old females who used alcohol (from 19.7% [95% CI, 18.0%-21.5%] to 16.7% [95% CI, 15.2%-18.4%]; *P* = .03). Moderate-to-severe AUD remained stable among females overall and across subgroups.

### Prevalence and Severity of CUD vs AUD Among Young Adults

Among 18- to 20-year-old individuals of both sexes in 2024, CUD prevalence exceeded AUD (14.5%-14.9% vs 10.3%-10.4%), despite higher prevalence of alcohol use than cannabis use (47.7%-50.5% vs 29.8%-30.7%). CUD prevalence among those who used cannabis was more than twice that of AUD among those who used alcohol (47.2%-50.0% vs 20.5%-21.7%).

Most males and females aged 18 to 20 years and males aged 21 to 34 years with CUD had moderate-to-severe CUD (51.0%-61.0% in 2024), a consistent pattern from 2022 through 2024. Most males and females with AUD—overall and across subgroups—presented with mild AUD during 2021-2024.

## Discussion

This cross-sectional study found divergent age- and sex-specific patterns from 2021 through 2024, with CUD and moderate-to-severe CUD prevalence increasing, while AUD and moderate-to-severe AUD prevalence remained stable or declined. The steepest increases in CUD and moderate-to-severe CUD prevalence occurred among both males and females aged 35 to 49 years and males aged 50 years or older, with a 7-fold increase in moderate-to-severe CUD among females aged 50 years or older. By contrast, AUD and moderate-to-severe AUD declined among males aged 21 to 34 years, while AUD declined among females aged 21 to 34 years and 35 to 49 years. This divergence may reflect behavioral and cultural shifts—including increasing cannabis preference over alcohol^4^—and declines in binge or heavy drinking.^3^

Findings repudiate conventional assumptions that cannabis carries a low addiction risk and that most young adults with CUD have mild symptoms. In 2024, past-year cannabis use was reported by 29.8% of males and 30.7% of females aged 18 to 20 years and by 37.4% of males aged 21 to 34 years; among those who used cannabis, 45.8% to 50.0% had CUD; and of those with CUD, 51.0% to 61.0% had moderate-to-severe CUD. For adults aged 18 to 20 years, despite higher prevalence of alcohol use than cannabis use, CUD prevalence among those who used cannabis was more than twice that of AUD among those who used alcohol; most individuals with AUD had mild AUD. Findings align with evidence on the greater addictiveness of high–delta-9-tetrahydrocannabinol (primary addictive component of cannabis) products and their widespread availability and acceptance.^5,6^ Prior research shows high CUD prevalence among young people within 12 months of initiation, persisting beyond 36 months.^7,8^

CUD is associated with adverse physical, cognitive, and psychosocial outcomes,^9^ including suicidality,^10^ violence,^4^ and schizophrenia (especially in young men).^11^ Moderate-to-severe CUD warrants clinical treatment. However, despite increasing prevalence of moderate-to-severe CUD, treatment rates have declined,^12^ alongside expanding cannabis legalization and increasing perceived benefits. Regardless of legalization status, physicians may recommend medical use,^13^ but medically recommended cannabis does not confer a reduced risk of moderate-to-severe CUD compared with nonmedical use.^14,15^

High prevalence of moderate-to-severe CUD underscores the importance of early detection, routine screening, timely access to and sustained engagement^15^ in evidence-based CUD treatment, and targeted strategies to counter increasing acceptance, availability, and potency of cannabis products. If clinicians recommend medical cannabis, making patients aware of risks across modes of cannabis consumption, assessing CUD risk, and implementing structured monitoring are warranted to detect early CUD signs. Unlike AUD,^1^ no US Food and Drug Administration–approved medications exist for CUD. Systems of care should ensure that patients receive timely education, screening, and intervention to mitigate CUD risk and address CUD and related consequences.

### Limitations

This study has several limitations. It may underestimate CUD and AUD prevalence and severity because NSDUH excluded unsheltered individuals and incarcerated populations. NSDUH is a self-report survey and is subject to social-desirability bias. Because we aimed to explore patterns in CUD, AUD, and severity over time, multiple-comparison corrections were not applied. Future research may examine how trends vary by state-level cannabis policy.

## Conclusions

Findings from this cross-sectional study suggest an urgent need for evidence-based, age- and sex-inclusive public health and clinical strategies to address rapidly evolving patterns of cannabis use, CUD, and moderate-to-severe CUD. More effective CUD treatments are needed. Increasing CUD prevalence and severity among adults who use cannabis challenge the notion that cannabis has low addictive potential.

## Notes

Supplement. Data Sharing Statement
