# Supplementary material for: Trends in the Prevalence and Severity of Alcohol and Cannabis Use Disorders Among US Adults
Source: JAMA Psychiatry. 2026 Aug 19;83(10):1056–61. doi: 10.1001/jamapsychiatry.2026.2497 (PMC13491236; doi:10.1001/jamapsychiatry.2026.2497)
Supplement: Supplementary file 1 [file jamapsychiatry-e262497-s001.pdf]

Data Sharing Statement

Han. Trends in the Prevalence and Severity of Alcohol and Cannabis Use Disorders Among US Adults. *JAMA Psychiatry*. Published August 19, 2026.
doi:10.1001/jamapsychiatry.2026.2497

Data
Data available: Yes
Data types: Deidentified participant data, Data dictionary
How to access data: Data and the data dictionary are available online.
When available: With publication

Supporting Documents
Document types: Other (please specify)
Additional Information: Data and the data dictionary are available online. The website address: https://www.samhsa.gov/data/data-we-collect/nsduh-national-survey-drug-use-and-health/datafiles
How to access documents: The website address: https://www.samhsa.gov/data/data-we-collect/nsduh-national-survey-drug-use-and-health/datafiles
When available: With publication

Additional Information
Who can access the data: Anyone requesting the data.
Types of analyses: For any purpose.
Mechanisms of data availability: Without investigator support.
Any additional restrictions: None.
